# Local Forest Structure and Host Specificity Influence Liana Community Composition in a Moist Central African Forest

**DOI:** 10.1002/ece3.71075

**Published:** 2025-03-11

**Authors:** Begüm Kaçamak, Maxime Réjou‐Méchain, Nick Rowe, Vivien Rossi, Nicolas Barbier, Samantha Bazan, Eric Forni, Daniel Guibal, David J. Harris, Grace Jopaul Loubota Panzou, Jean‐Joël Loumeto, Eric Marcon, Bruno X. Pinho, Isaac Zombo, Sylvie Gourlet‐Fleury

**Affiliations:** ^1^ AMAP University of Montpellier, IRD, CNRS, CIRAD, INRAE Montpellier France; ^2^ Cirad UPR Forêts et Sociétés Montpellier France; ^3^ Remote Sensing and Forest Ecology Lab, Higher Teacher's Training College University of Marien Ngouabi Brazzaville Republic of the Congo; ^4^ Cirad UMR Selmet Montpellier France; ^5^ Cirad‐PERSYST‐UPR BioWooEB Montpellier France; ^6^ Royal Botanic Garden Edinburgh UK; ^7^ Institut Supérieur Des Sciences Géographiques, Environnementales et de l'Aménagement (ISSGEA) Université Denis Sassou Nguesso Kintélé Republic of the Congo; ^8^ Faculté Des Sciences et Techniques, Laboratoire de Biodiversité, de Gestion Des Ecosystèmes et de l'Environnement (LBGE) Université Marien Ngouabi Brazzaville Republic of the Congo; ^9^ AgroParisTech Montpellier France; ^10^ Institute of Plant Sciences University of Bern Bern Switzerland; ^11^ CIB‐Olam Ouesso Republic of the Congo

**Keywords:** Congo Basin, determinants of plant community diversity and structure, ecological strategies, functional traits, tropical forests, wood density, woody vines

## Abstract

Lianas are important components of tropical forest diversity and dynamics, yet little is known about the drivers of their community structure and composition. Combining extensive field and LiDAR data, we investigated the influence of local topography, forest structure, and tree composition on liana community structure, and their floristic and functional composition, in a moist forest in northern Republic of Congo. We inventoried all lianas ≥ 1 cm in diameter in 144 20 × 20‐m quadrats located in four 9‐ha permanent plots, where trees and giant herbs were inventoried. We characterized the functional strategies of selected representatives of the main liana taxa using a set of resource‐use leaf and wood traits. Finally, we used complementary statistical analyses, including multivariate and randomization approaches, to test whether forest structure, topography, and tree composition influence the structure, floristic composition, and functional composition of liana communities. The structure of liana communities was strongly shaped by local forest structure, with higher abundances and total basal areas in relatively open‐canopy forests, where lianas competed with giant herbs. Liana floristic composition exhibited a weak spatial structure over the study site but was marginally influenced by the local forest structure and topography. Only forest structure had a weak but significant effect on liana functional composition, with more conservative strategies—higher stem tissue density and lower PO_4_ leaf concentration and SLA values—in tall and dense forests. Finally, we found evidence of host specificity with significant attraction/repulsion for 19% of the tested liana and tree species associations, suggesting that the unexplained floristic variation may be partly attributed to these host‐species‐specific associations, although the underlying mechanisms behind remain elusive. Overall, our findings demonstrate that liana communities' structure can be much better predicted than their composition, calling for a better understanding of the implications of the large functional diversity observed in liana communities.

## Graphical abstract

Our study unravels liana community dynamics in Congo, addressing the scarcity of central African liana literature. Using unique datasets, we offer original insights into liana functional ecology, host specificity, and competition with herbs.

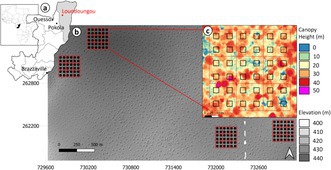

## 1 Introduction

Lianas are increasingly recognized as major players in tropical forest dynamics (Marshall et al. 2020; Laurance et al. 2014; Tang et al. 2012; Schnitzer and Bongers 2005; Schnitzer and Bongers 2002; Putz 1984a, 1984b). They can contribute up to 40% of plant diversity and 25% of plant density (Schnitzer and Bongers 2002) and significantly alter the energy balance and carbon cycling of tropical forests (Porcia e Brugnera et al. 2019). They are known to negatively affect tree growth, survival, and regeneration through above‐ and below‐ground competition, thus reducing tree diversity and forest carbon storage (Ingwell et al. 2010; Laurance et al. 2014; Phillips et al. 2005; Schnitzer et al. 2005, 2014a, 2014b; Schnitzer and Carson 2010; van der Heijden et al. 2015; Van der Heijden and Phillips 2009). Most recent liana studies have focused on their effects on forest dynamics, considering them as a single functional plant type (e.g., Ingwell et al. 2010; Laurance et al. 2014; Porcia e Brugnera et al. 2019). However, as noted nearly 50 years ago (Jacobs 1976), liana ecology remains “largely unknown” (Schnitzer et al. 2014a, 2014b). Lianas occur in more than 133 angiosperm families (Gentry 1991) and have recently been shown to exhibit great variability in life‐history strategies, with a range of functional traits at least as great as that of trees (Medina‐Vega et al. 2021; Meunier et al. 2020; Sun et al. 2022). Resource partitioning among different liana species with different ecological preferences is thus likely important but remains poorly documented (Liu et al. 2021a, 2021b). This limits our ability to understand and predict current and future tropical forest dynamics (Marshall et al. 2020).

It has been hypothesized that the structure of liana communities is closely related to the local forest structure (Ledo and Schnitzer 2014). Indeed, lianas require external physical supports to reach the canopy and are therefore expected to depend primarily on surrounding forest structure (Balfour and Bond 1993; Hegarty and Caballé 1991). Liana abundance has been consistently shown to vary along gradients of forest stand structure, mostly through a decrease in liana abundance with increasing canopy height (Dalling et al. 2012; Villagra et al. 2021). Also, forest disturbance is known to be a major driver of liana abundance (Ledo and Schnitzer 2014; Schnitzer et al. 2021; Villagra et al. 2021) because most species have a high clonal reproduction rate that allows them to rapidly invade disturbed areas in canopy gaps (Dalling et al. 2012; Ledo and Schnitzer 2014; Schnitzer et al. 2012, 2021; Waite et al. 2023). However, a recent study conducted in Thailand showed that both liana abundance and basal area increased significantly along a forest successional gradient (Lomwong et al. 2023). This result was inconsistent with previous studies mostly conducted in central and south America where liana abundance declined systematically with forest stand age (Letcher and Chazdon 2009; Dewalt et al. 2000) and potentially suggests important biogeographical differences in liana ecology. Abiotic factors, such as topography and soil conditions, also influence liana community structure. For instance, the abundance of lianas tends to be higher in low elevation areas (Addo‐Fordjour and Rahmad 2015; Bruy et al. 2017; Schnitzer et al. 2012), in forests with a shallow water table (Gerolamo et al. 2018), and in nutrient‐rich soils (Addo‐Fordjour and Rahmad 2015; DeWalt et al. 2006; Gerolamo et al. 2018; Lai et al. 2017). A recent study in Asia even showed that soil properties have a stronger influence on liana abundance than forest structure (Liu et al. 2021a, 2021b). This contrasts with previous findings from Neotropical forests, where richer soils were either less or not significantly associated with liana abundance (Dalling et al. 2012; Schnitzer et al. 2020). Thus, the importance of forest structure and abiotic conditions on the local liana community structure may be less clear than previously assumed.

The local drivers of the taxonomic and functional composition of liana communities were far less studied than their structure, in part, due to difficulties in plant identification and taxonomic ambiguities (Schnitzer and Bongers 2002). Lianas were up to now usually considered as light‐demanding, or more generally resource‐acquisitive, species, as compared to trees (Collins et al. 2016). However, their ecological strategies are known to widely differ among families (Putz and Mooney 1991) and a few studies already demonstrated that the large interspecific variance in light requirements, and more generally in functional strategies, resulted in important liana compositional turnover along ecological and environmental gradients (Dalling et al. 2012; DeWalt et al. 2006; Liu et al. 2021a, 2021b; Lomwong et al. 2023; Mumbanza et al. 2020; Rocha et al. 2022; Yuan et al. 2016). For instance, liana species composition has been found to differ significantly among forest types (Mumbanza et al. 2020), and different liana species were shown to dominate at different stages of forest succession (Lomwong et al. 2023), with a dominance of resource‐acquisitive liana taxa, e.g., with low wood density and high leaf nutrient concentration, in open canopy and young forests, and more conservative strategies in tall and mature forests (Mumbanza et al. 2022; Liu et al. 2021a, 2021b; Villagra et al. 2021). Liana floristic and functional compositions were also shown to be influenced by abiotic factors such as topography (Dalling et al. 2012) or soil properties (Chanthorn et al. 2024; DeWalt et al. 2006). For instance, in central Amazonia, a higher representation of liana taxa with low specific leaf area (SLA) and wood densities was found in valleys, while forest disturbances had a weaker influence on liana composition (Rocha et al. 2022). Despite recent advances in liana ecology, we still need to further explore the different life‐history strategies of lianas and to understand how they vary under different local environmental conditions.

Because lianas physically depend on tree support and exhibit a wide range of climbing strategies, they closely interact with tree architecture and morphology (Hegarty 1991). Therefore, the floristic composition of lianas may also be linked to the floristic composition of trees through host specificity, regardless of the forest structure and abiotic environment. To date, host prevalence has been mostly studied from a tree perspective, with liana infestation probability depending on tree species identity, architecture, or morphology, but not on liana identity (Campanello et al. 2007; Carsten et al. 2002; Heijden et al. 2008; Loubota Panzou et al. 2022; Nesheim and Økland 2007; Pérez‐Salicrup et al. 2001). The few studies that have examined species‐specific associations between lianas and trees considering liana species identity have led to contrasting results. While some have found host‐specific relationships for the main dominant liana species in their study site (Heymann et al. 2022; Song et al. 2023; Uwalaka et al. 2021), others showed no host‐specific relationships between tree species and the dominant liana species, and no relationships between functional morphological traits of trees and lianas (Vivek and Parthasarathy 2016). To date, there is thus no consensus on whether liana species exhibit tree host specificity. While some studies provided evidence for non‐random associations between lianas and trees, with varying levels of specificity (Addo‐Fordjour et al. 2021; Addo‐Fordjour and Afram 2021; Sfair et al. 2010), others have also shown a general random association of lianas and tree species (Addo‐Fordjour and Afram 2021; Ofosu‐Bamfo et al. 2022; Wilson et al. 2024). This information is needed to better understand liana colonization strategies, unravel mechanisms of potential host selectivity, and better predict liana–tree dynamics over time and space.

Here, we examined the distribution patterns of liana community structure, as well as their floristic and functional composition, using an extensive liana inventory (144 20 × 20‐m quadrats) from a moist tropical forest in northern Republic of Congo—an area that is scientifically underexplored (Sosef et al. 2017). We combined dendrometric measurements with various functional characteristics of leaves and stems of lianas, as well as drone‐based measurements (stereophotogrammetry and LiDAR) to characterize the local environment. We aimed to answer the following main question and the three hypotheses derived from it (Hn): To what extent do forest structure and tree composition influence the structure, floristic, and functional composition of liana communities?

*Liana structure: we expect higher densities of small liana stems in relatively short‐stature and open‐canopy forests*.

*Floristic and functional compositions of lianas: we expect that the floristic composition of lianas is significantly influenced by forest structure with a higher representation of resource‐acquisitive taxa in shorter stature and more open canopies*.

*Host specificity: we expect covariation in the floristic composition of trees and lianas due to common environmental factors rather than host‐species specificity*.

## 2 Materials and Methods

### 2.1 Study Site

**FIGURE 1:**
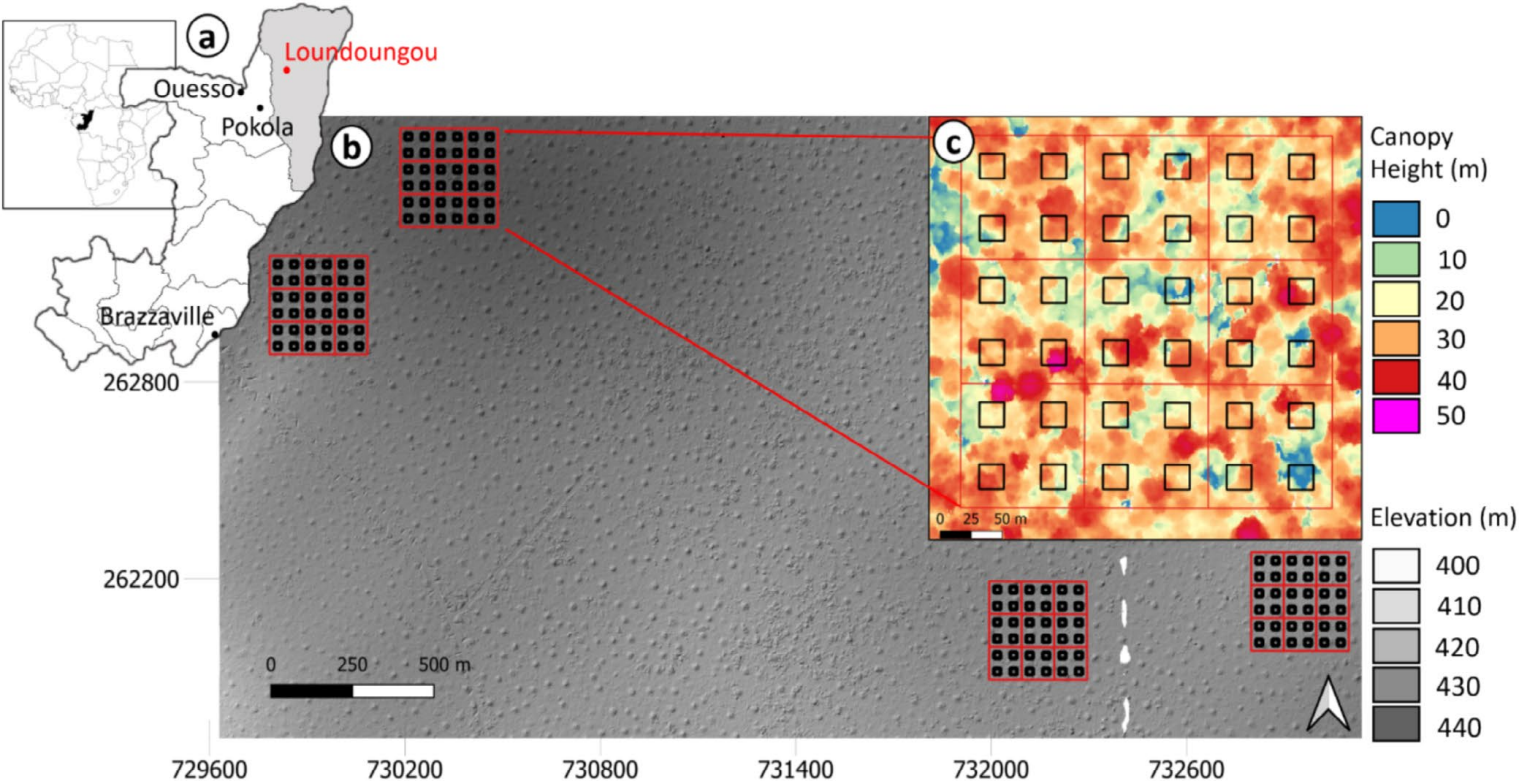
Study site and experimental design. (a) The Loundoungou site (in red) is located in the Likouala province (in gray) in the Republic of Congo (in black). (b) The four 9‐ha plots where trees were measured are overlaid on a 1‐m hillshaded digital elevation model derived from LiDAR data. It specifically illustrates the high abundance of large termite mounds and the homogeneity of the site topography. (c) A zoom of one 9‐ha plot overlaid on a LiDAR‐derived canopy height model showing the sampling design of the 20 × 20 m liana quadrats (in black).

The study area is located in the Likouala Province in the north of the Republic of Congo (2°27′11.87″N and 17°02′32.17″E; Figure 1). The mean annual rainfall is 1605 mm/year (unpublished data from 2003 to 2017 obtained from the nearest station in Pokola, 140 km from the study area), with two dry seasons from June to August and from December to February. The site is located on a plateau with an elevation of 395–470 m a.s.l. and a geological substrate of Quaternary limestone and alluvium. The soil type is classified as Xanthic Acrisols and shows little textural variation (from sandy‐loamy to loamy‐sandy) between the highest (470 m) and lowest (395 m) elevations of the plateau (Freycon 2014). The vegetation corresponds to the semi‐deciduous forest type, except for a few patches of monodominant *Gilbertiodendron dewevrei* (Caesalpiniaceae) forests, with a large number of trees belonging to the Euphorbiaceae (20%), Fabaceae (12%), Meliaceae (9%), Ebenaceae (8%), and Annonaceae (7%) families (Forni et al. 2019; Réjou‐Méchain et al. 2021). Little is known about human history in the area, but there is no evidence of recent human activity (< 100 years), except for hunting and a recent, low‐intensity logging (0.3 trees/ha) that occurred in the late 2018, after the liana inventory and the drone acquisitions (see below).

### 2.2 Liana and Tree Inventory Data

The experimental site was first established in 2014 in the Loundoungou forest management unit (UFA), conceded to the Olam‐Agri logging company, in Likouala Province. The experimental design consists of four permanent 9‐ha plots (300 × 300 m) where all trees ≥ 10 cm DBH (diameter at breast height) were marked, identified, and positioned and their diameters were measured every year since 2015 according to international standards (Picard and Gourlet‐Fleury 2008). To compare different silvicultural treatments, the experimental design of the plots was originally designed to minimize the effects of abiotic conditions, with a maximum elevation difference of only 23 m between plots (Figure 1).

Liana inventories were conducted from April to July 2017 within 144 20 × 20‐m quadrats (total area of 5.76 ha) established at regular intervals within the permanent plots (4 quadrats/ha; Figure 1). All woody lianas and rattans ≥ 1 cm in diameter were marked with paint, and their diameters were measured at 1.3 m from their rooting point, according to international protocols (Gerwing et al. 2006; Schnitzer et al. 2008). For each liana, the host tree and, if applicable, the identity of the second host tree were noted to assess whether lianas display host specificity (see Section 2.5.2.). Given the lack of expertise in liana taxonomy in this region, lianas were first identified using vernacular names in a regional language, Enyellé, from the Likouala region (Pandzou). Because a vernacular name may represent more than one species or a species may have multiple vernacular names at different ontogenetic stages, we conducted complementary work to assess the taxonomic coherence of vernacular names and, when possible, assigned botanical names to lianas. To this end, we collected leaf samples in May 2018 from 284 lianas identified by the same fieldworkers as in 2017. These samples belonged to 64 out of the 82 vernacular names inventoried in 2017, but these 64 vernacular names represented 98% of the liana individuals in the dataset. Leaf samples were collected primarily in and around the four 9‐ha plots in shaded locations where lianas had sprouted yielding leafy shoots, maximizing the number of samples for the most abundant lianas (up to 17 samples per vernacular name; 4 samples on average). Two herbarium specimens were collected from each individual, one deposited in the ALF herbarium at Cirad in Montpellier (Bazan 2021) and the other in the national Forest Research Institute herbarium in Brazzaville. A subset of the leaf material was dried with zeolite beads in ziplock bags for genetic analyses (see Appendix S1: Supplementary 1) to identify taxonomic groups, verify taxonomic coherence, and validate the scientific names of the lianas. 57.7% of the lianas from the inventory were identified at the species level, 9.2% at the morphospecies level, 30.8% at the genus level, 0.5% at the family level, and we were unable to identify 1.8% of all lianas.

### 2.3 Liana Functional Traits

We measured functional leaf and stem traits that position species along the “fast–slow” plant‐economic spectrum (Reich 2014) for 44 liana taxa representing 95% of the liana stems measured in the inventory (range of 67%–100% among plots; Table 1). Leaf functional traits were measured on liana herbarium samples collected in May 2018, following the protocols and recommendations of Perez et al. (2020) for SLA and leaf thickness. These authors showed that values derived from herbarium measurements for SLA and thickness correlated strongly with values measured from fresh tissue, although values derived from herbarium SLA are slightly overestimated globally. SLA measurements were made with the petioles. After measuring mass and scanning all samples, we used imagej software (Abràmoff et al. 2004) and a semi‐automated method developed by Borianne and Brunel (2012) to measure leaf area. For leaf thickness measurements, we rehydrated leaves in a 1:50 solution of neutral household detergent and water for 20 min before measurements. Measurements were then taken at three points in the center of the leaf using a digital micrometer (Mitutoyo QuantuMike with a 0.001 mm of resolution), avoiding the leaf veins. In addition, we performed elemental analyses of carbon, nitrogen, phosphorus, and potassium of the herbarium liana leaves. We collected 100 mg of dry leaves per sample and ground them into a powder. For the elemental analyses of carbon and nitrogen (%C and %N, respectively), we used the Flash Smart AE 2000 elemental analyzer, which performs adsorption chromatography based on the Dumas method (Dumas 1831). For phosphorus and potassium, we first mineralized the samples and then determined the total phosphorus (%PO4) by colorimetric determination using molybdenum blue with the HNO3 gallery and the potassium concentration (%K) using a Thermo ICE 3000 Series atomic absorption spectrometer in flame mode.

**TABLE 1:** Description of leaf and stem liana functional traits used for 44 species.

Trait	Abb.	Range	Unit	Mean ind/taxa	Functional role
Leaf thickness	Thick	[0.04–0.2]	mm	5	Leaf structure mechanical support and defense
Specific leaf area	SLA	[13.0–64.7]	mm^2^ mg^−1^	5	Light capture and growth: leaf longevity, photosynthetic rate
Leaf Carbon	%C	[38.9–48.2]	%	5	Leaf investment on structures
Leaf Nitrogen	%N	[2.1–5.8]	%	5	Light capture and growth: carbon fixation (photosynthesis and respiration)
Leaf Phosphorus	%PO_4_	[0.07–0.27]	%	5	Light capture and growth: enzyme function
Leaf Potassium	%K	[0.3–2.2]	%	5	Maintenance metabolism: membrane function
Stem Tissue density	TD	[0.16–0.59]	g.cm^3^	4	Stem transport, mechanical structure, longevity, and defense against herbivory

We additionally measured liana wood densities on liana stem samples. In May 2018 and October 2021, we collected 211 stem cylinders above 2 cm in diameter (range 2–10 cm) at 1.3 m from the stem root using an opportunistic sampling method outside the permanent plots to avoid destructive measurements within the plots. Stem samples were debarked, keeping their pith, and saturated with water under pressure for 48 h before measuring Archimedes thrust, to estimate the volume of the stem sample (equivalent to the mass of the volume of water moved when immersed), according to the protocol described by Birouste et al. (2014). The samples were then first dried in an oven at 40°C for 10 days to avoid damaging the water‐saturated materials with extreme temperatures. We then increased the oven temperature to 103°C for 48 h to remove all water residues. Finally, the anhydrous masses of the wood samples were measured to determine the wood density, hereafter referred to as stem tissue density (TD) because the stems of lianas, even when debarked, are not simple wood cylinders like trees, but most species have a mixture of different tissues consisting of both secondary xylem (wood) and other soft and lignified primary and secondary tissues.

### 2.4 Forest Structure and Topography

To characterize the environment, we considered four groups of variables: ground‐based tree variables, drone‐based canopy variables, ground‐based giant herb variables, and drone‐based topographical variables. We assigned five ground‐based tree variables to each 20 × 20‐m liana quadrat with a 10‐m buffer on each side (40 × 40‐m subplots) because trees in close proximity to the liana quadrats may also influence liana structure and composition. We used the 2017 tree inventory dataset to calculate four stand‐level measures: tree stem density, called tree abundance hereafter (N_T_ in stems/ha), tree quadratic mean diameter (QMD_T_ in cm), tree total basal area (BA_T_ in m^2^/ha), and tree mean wood density (WD_T_ in g/cm^3^). Tree wood density values were extracted from the global wood density database (Chave et al. 2009; Zanne et al. 2009) through a procedure implemented in the BIOMASS package (Réjou‐Méchain et al. 2017). The fifth ground‐based tree variable corresponded to the basal area change, ΔBA_T_ (in m^2^), between the 2015 and 2017 inventory (ΔBA_T_ = BA_T2017_‐BA_T2015_), also calculated at the 40 × 40‐m scale.

We measured canopy‐level variables using drone imagery (see details on the acquisitions in Kaçamak et al. (2022)). Briefly, red‐green‐blue (RGB) images were acquired in June 2018 and used to create a digital surface model (DSM_2018_) at a 1‐m resolution using a stereophotogrammetry approach. Thanks to a LiDAR‐derived digital elevation model acquired in February 2020 (DEM_2020_), we created a canopy height model (CHM_2018_) by subtracting DEM_2020_ from DSM_2018_. Finally, we used CHM_2018_ to calculate the mean of the top of the canopy height (meanTCH in m) at the quadrat level with a 10‐m buffer on each side (40 × 40‐m subplots), as done for the ground‐based tree variables. A high meanTCH typically represents a close and high canopy, while a low meanTCH represents an open canopy, e.g., due to tree gaps.

Because lianas can interact with life forms other than trees, giant herbs from the orders Commelinales and Zingiberales were inventoried in the four 9‐ha plots. These giant herbs are widespread in lowland forests of central and western Africa (Pouteau et al. 2024), particularly at our study site where *Haumania danckelmaniana* (J. Brun et K. Schum.) Miln. Redh (Marantaceae), a climbing form, dominates. The inventory was conducted on a systematic 10‐m grid, resulting in 100 sampling points per hectare. At each sampling point, we quantified the projected leaf cover of giant herbs on the ground using a semi‐continuous index from 1 to 3, where 1 represents low leaf cover (< 20%), 2 represents leaf cover between 20% and 70%, and 3 represents high leaf cover (> 70%). Therefore, we calculated the mean leaf cover of giant herbs over liana quadrats (GHFC) as the mean of the nine sampling points within the quadrat.

Finally, as the topography of the study area was homogeneous (Figure 1), we measured only local topographic variations due to the presence of numerous large termite mounds that may have an impact on water and nutrient availability (1.5–3 large mounds per ha with a radius of 9.9 ± 3.3 m and a height of 4.1 ± 1.5 m; see Appendix S1: Supplementary 2; Freycon et al. 2018; Penel et al. 2022). We used DEM_2020_ and the SAGA module of QGIS version 3.10 to derive the relative slope position index (RSP; see Appendix S1: Supplementary 3) to characterize termite mounds at the quadrat level. The RSP is measured as follows:

$$\mathrm{RSP}=\frac{\text{AACL}}{\text{AACL}+\text{ABRL}}$$

with AACL being the altitude above channel lines and ABRL being the altitude below ridges (Bock et al. 1996). Hence, high RSP values (RSP = 1) represent the top of the termite mounds (see Appendix S1: Supplementary 3).

All the studied environmental variables displayed correlations < 0.7 with each other.

### 2.5 Statistical Analyses

**FIGURE 2:**
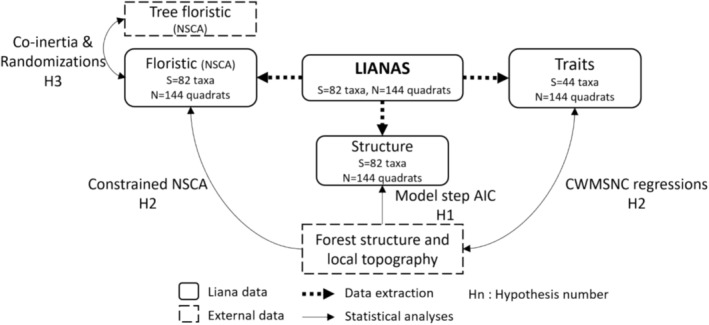
Diagram illustrating the different statistical analyses used to test the study hypotheses (Hn, see the last paragraph of the introduction). AIC, Akaike Information Criterion; CWMSNC, Community Weighted Mean Species Niche Centroid; NSCA, Non‐Symmetric Correspondence Analysis; PCA, Principal Component Analysis. The arrows representing the statistical analyses are unidirectional when the model tests the effect of one variable on another and bidirectional when the model explores the co‐structure between two variables without specifying one as the explanatory variable.

The analyses performed on the structure and floristic composition of liana communities were based on the entire liana inventory dataset, whereas the analyses of the functional composition were conducted with the subset of 44 liana taxa for which functional traits were measured (95% of individuals in the liana inventory). All analyses described below are summarized in Figure 2 and were performed using the statistical software R 4.2.1 (R Core Team 2022).

#### 2.5.1 Drivers of Liana Community Structure

To test the relationship between the structure of liana communities and environmental gradients, we first calculated three liana metrics at the 20‐m quadrat level: liana abundance, defined as the number of stems standardized to the 1‐ha scale (N_L_ in stems/ha), total basal area (BA_L_ in m^2^/ha), and quadratic mean diameter (QMD_L_ in cm) of lianas. We then used linear models and a model selection procedure to identify the main environmental factors affecting liana structure. Because lianas were inventoried in a nested sampling design (quadrats in 1‐ha subplots and 1‐ha subplots in 9‐ha plots), our observations were not independent. Therefore, we first tested a possible pseudo‐replication effect in our dataset, using linear mixed models. The effects of the 1‐ha or 9‐ha plots were subsequently accounted for in a random intercept using the restricted maximum likelihood (REML) approach. We performed likelihood ratio tests (LR) to test the statistical significance of the random term (Bolker et al. 2009). Because the random effect was not significant in any models (*p*‐value > 0.1; results not shown), we performed all analyses using only the fixed effects (our predictors) for the sake of model parsimony, after scaling all explanatory variables. We finally selected the model with the lowest Akaike Information Criterion (AIC) for each liana structure variable using a stepwise forward and backward selection approach.

#### 2.5.2 Drivers of Liana Floristic Composition

To characterize the floristic composition of the liana community, we initially performed a correspondence analysis and found that the results were highly influenced by some very rare species. Therefore, we additionally performed a non‐symmetric correspondence analysis (NSCA), which is similar to a correspondence analysis but gives more weight to abundant species (Pélissier et al. 2003). We then tested whether the floristic composition of lianas at our study scale was spatially structured by performing permutation tests for spatial variograms (*n* = 9999 random distributions; Diblasi and Bowman 2001), using the site scores on the first two NSCA axes. This test indicates whether the spatial variogram deviates significantly from a random structure at a given distance class and thus shows whether spatial, and thus potentially confounding, drivers are in play in the floristic composition.

For consistency with our previous analysis, we assessed the effect of the environment on the floristic composition of lianas using a constrained ordination (NSCAIV, i.e., non‐symmetric canonical correspondence analysis; Couteron et al. 2003) with all environmental variables (H2, Figure 2). NSCAIV is similar to a canonical correspondence analysis (CCA) but, again, gives more importance to abundant species as done in a redundancy analysis (Pélissier et al. 2003). To assess the effect of the environment on floristic composition while controlling for potential spatial dependencies, we performed a partial canonical correspondence analysis (PCCA) using a second‐order polynomial function of the quadrat coordinates as a conditional table. For both analyses, we employed permutation tests (*n* = 999 randomizations) to determine whether the explanatory variance of the environmental variables was significant.

#### 2.5.3 Drivers of Liana Functional Composition

We examined liana trait–environment relationships using an approach based on the combination of two parametric tests at the community and species level, known as Community Weighted Mean Species Niche Centroid regressions (hereafter referred to as CWM/SNC regressions), as recently introduced by ter Braak (2019), TraitEnvMLMWA package by ter Braak (2022). This is a novel powerful regression‐based analysis that accounts for the fourth‐corner problem (Peres‐Neto et al. 2017) and allows assessing the influence of environmental gradients on community functional composition (i.e., CWM traits) in combination with how species distribution, parameterized by species niche centroids (SNC) in environmental space, relates to their traits. CWM and SNC regressions were weighted by Hill numbers of order 2 (i.e., N_2_‐weighted; ter Braak 2019), which gives more importance to abundant species. In this method, *p*‐values are obtained by permutation of sites in CWM‐regression and species in SNC‐regression, and the final *p*‐value is equal to the maximum *p*‐value of the two tests (ter Braak et al. 2018). Therefore, a significant trait–environmental relationship reflects not only changes in trait dominance across communities, which could be driven by few dominant species, but rather a consistent response to the environment across species with similar traits (Lepš and De Bello 2023).

We further examined whether the main floristic gradients, represented by the two first NSCA axes, were associated with significant changes in functional composition. We specifically randomized liana functional traits among species (*n* = 999) to build null distributions of Pearson's correlation values between CWM traits and NSCA scores.

#### 2.5.4 Host Specificity

We examined whether the floristic composition of the liana and tree communities significantly covaries with each other by performing a Monte Carlo test on the sum of eigenvalues of a co‐inertia analysis, based on the R_V_ coefficient (Dray et al. 2003; Heo and Gabriel 1997; H2b). To test whether covariation between the floristic composition of the liana and tree communities arises only because of a common response to environmental gradients, we also performed this analysis on the residuals of NSCAIVs applied to both liana and tree communities, i.e., assessing covariation between liana and tree floristic variations that are unexplained by the studied environmental variables.

We tested the existence of host‐species specificity using randomization tests of liana and tree associations. We restricted this analysis to lianas climbing into an identified tree (*n* = 2737 lianas) and kept the identity of the second host tree if present (considering the first host tree only resulted in marginal changes). At each randomization step (*n* = 999), we randomly assigned a tree within the quadrat (including the 10‐m buffer around each quadrat) to each liana individual that occurred in the same quadrat to create a null dataset corresponding to the expected number of infested trees per tree species for each liana taxon obtained by chance alone. Restricting this randomization process to the quadrat level allowed us to account for spatial autocorrelation in both the distribution of tree and liana taxa and thus to limit the potential influence of common environmental drivers. Finally, we compared the observed number of individual lianas infesting each tree species to the expected null distribution using the 2.5th and 97.5th percentiles to detect significant repulsion or attraction between lianas and tree taxa. To ensure statistical power, we limited our analyses to the 10 most abundant liana taxa (> 60 individuals per taxa in the entire data set, for a total of 2110 liana individuals) and reported results only for tree species with more than 100 individuals in the entire dataset (*n* = 24 species). We also recorded the climbing mechanisms of the 10 most abundant liana taxa to investigate whether climbing mechanisms play a role in host‐species specificity. We classified climbing mechanisms according to Sperotto et al. (2020). Twining lianas with tendrils, prehensible branches, or angular branches were classified as “active” climbing mechanisms, while scrambling lianas with hooks, spines, or adhesive roots were classified as “passive” climbing mechanisms.

## 3 Results

### 3.1 Liana Community Structure

A total of 6206 liana stems ≥ 1 cm in diameter were recorded, corresponding to a density of 1077 stems per hectare. Overall, liana communities were dominated by small‐size individuals, with 62% of stems between 1 and 2 cm and 94% between 1 and 5 cm, with a maximum diameter of 27 cm at 1.3 m above their rooting point.

The best predictive models selected for each liana community structure variable are summarized in Table 2. Forest structure and dynamics appeared to have a strong influence on liana community structure. Liana abundance (N_L_) was predominantly negatively influenced by tree quadratic mean diameter (QMD_T_), mean canopy height (meanTCH), and tree mean wood density (WD_T_). Additionally, tree basal area change (ΔBA_T_) and giant herb foliar cover (GHFC) had a smaller negative effect (about half as important) on N_L_. Liana basal area (BA_L_) was driven by the same variables except for the absence of a significant effect of QMD_T_ and a more predominant effect of meanTCH whose effect was at least twice as high as the other variables. Finally, liana quadratic mean diameter (QMD_L_) tended to be influenced by a different set of explanatory variables with a positive effect from the tree total basal area (BA_T_) and WD_T_ and a negative effect from tree abundance (N_T_), all of comparable magnitude. The topographic variable associated with termites (RSP) was not selected for and did not appear to affect liana stand structure. Overall, liana abundance was the best‐explained dependent variable (38%), followed by total basal area of lianas (25%) and liana quadratic mean diameter (23%).

**TABLE 2:** Estimates of predictors in the selected models predicting the structure of liana communities in response to the local environment, with confidence intervals in parenthesis.

	Intercept	QMD_T_	MeanTCH	WD_T_	ΔBA_T_	GHFC	BA_T_	N_T_	*R* ^2^	*F* stat
N_L_	1077.4 (1011, 1144)	−172.353 (−256.24, −88.47)	−164.667 (−246.16, −88.47)	−151.524 (−228.51, −74.54)	−66.193 (−141.12, 8.73)	−81.271 (−158.98, −3.56)	—	—	0.38	16.8
BA_L_	0.62 (0.57, 0.67)	—	−0.140 (−0.19, −0.09)	−0.080 (−0.13, −0.03)	−0.103 (−0.16, −0.05)	−0.083 (−0.14, −0.03)	—	—	0.25	11.8
QMD_L_	0.48 (0.46, 0.51)	—	—	0.043 (0.013, 0.072)	—	—	0.078 (0.048, 0.11)	−0.061 (−0.09, −0.03)	0.23	14.1

*Note:* Parameters associated with variables that were not selected by the AIC step approach are not reported. For liana metrics, BA_L_, liana basal area; N_L_, liana abundance; QMD_L_, liana quadratic mean diameter. For environmental variables, BA_T_, tree total basal area; GHFC, giant herbs foliar cover; meanTCH, mean canopy height; N_T_, tree abundance; QMD_T_, tree quadratic mean diameter; WD_T_, tree mean wood density; ΔBA_T_, tree basal area change.

### 3.2 Liana Floristic Composition

The inventoried lianas belonged to 82 vernacular taxa and 22 families. The most abundant families were Euphorbiaceae (31%), Fabaceae (17%), Apocynaceae (13%), Loganiaceae (7%), and Connaraceae (5%). The liana communities were dominated by three hyperdominant taxa that accounted for 54% of the liana stem abundance (*Manniophyton fulvum* Muell.Arg., 30%; *Dalhousiea africana* S. Moore, 12%; and *Agelaea spp*. Sol ex. Planch, 12%).

**FIGURE 3:**
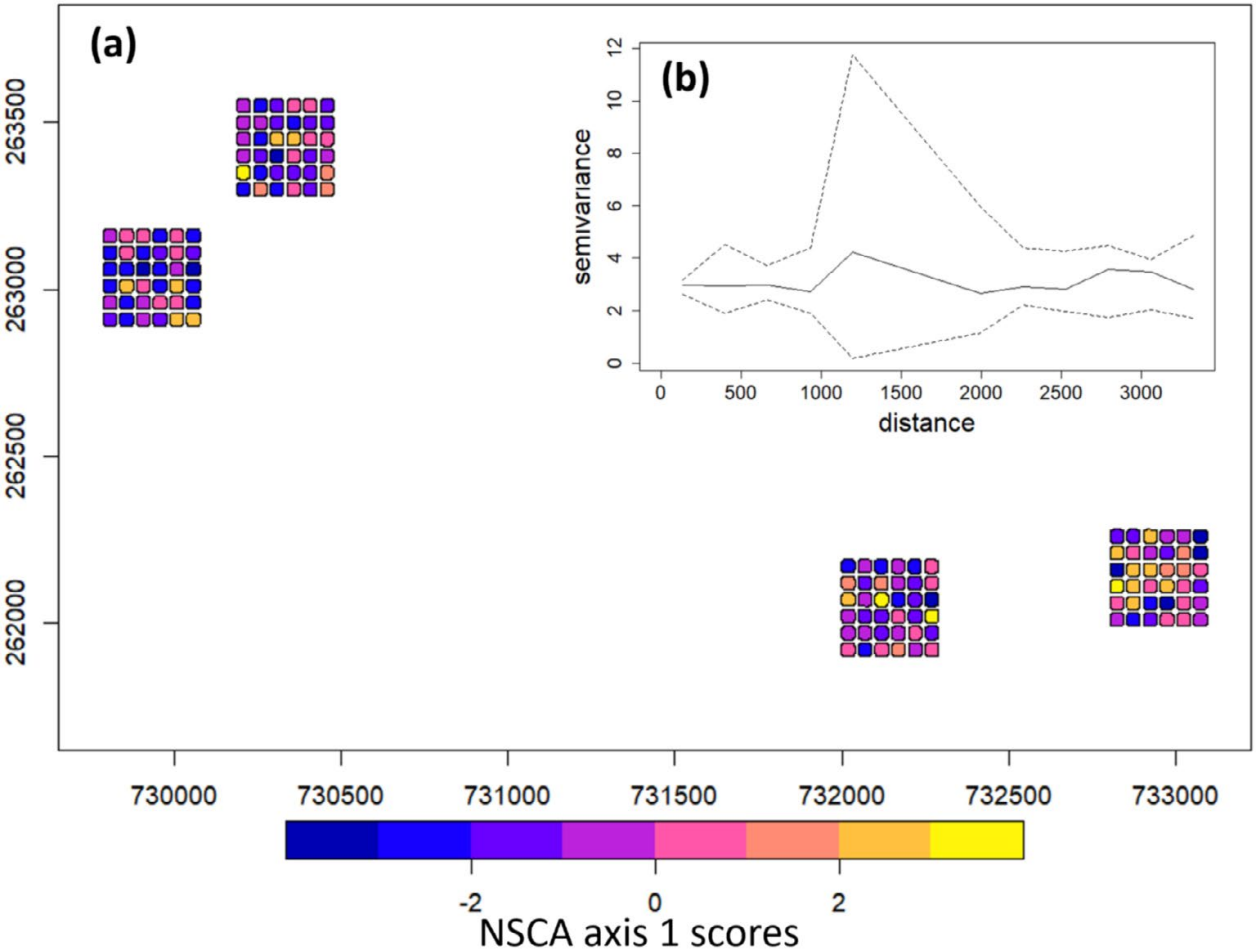
Spatial structure and floristic composition in the 144 liana quadrats and their spatial variogram. (a) Spatial distribution of the color‐coded scores of the first axis of the Non‐Symmetric Correspondence Analysis (NSCA) of liana floristic composition in UTM zone 33 N coordinates. There is no visible spatial pattern of the floristic composition of lianas (see different colors). For illustrative purposes, a 10‐m buffer has been added to the quadrat to better visualize the colors. (b) Inset graph representing the variogram of NSCA axis 1 scores, with 11 distance classes in the *x* axis and the variance in *y* axis (See Appendix S1: Supplementary 5 for the axis 2). The dotted lines on the variogram represent the confidence intervals at 95% and the solid lines the observed variogram. The variogram stays inside the confidence interval for all distance classes and does not deviate from a random structure, confirming the absence of a spatial pattern of liana floristic composition. Individual P‐values for each distance class are reported in Appendix S1: Supplementary 6.

The first two axes of the NSCA (see Appendix S1: Supplementary 4) were largely determined by these three most abundant taxa and captured 56% of the overall variation in the floristic composition of the lianas. As shown in Figure 3, the floristic composition of the liana community was not spatially structured at our study scale. Permutation tests performed on spatial variograms of the first two axes confirmed the lack of spatial correlation in all distance classes (Figure 3 for the first axis, see Appendix S1: Supplementary 5 for the second axis). Thus, floristic composition appeared to vary only locally within our study area.

**FIGURE 4:**
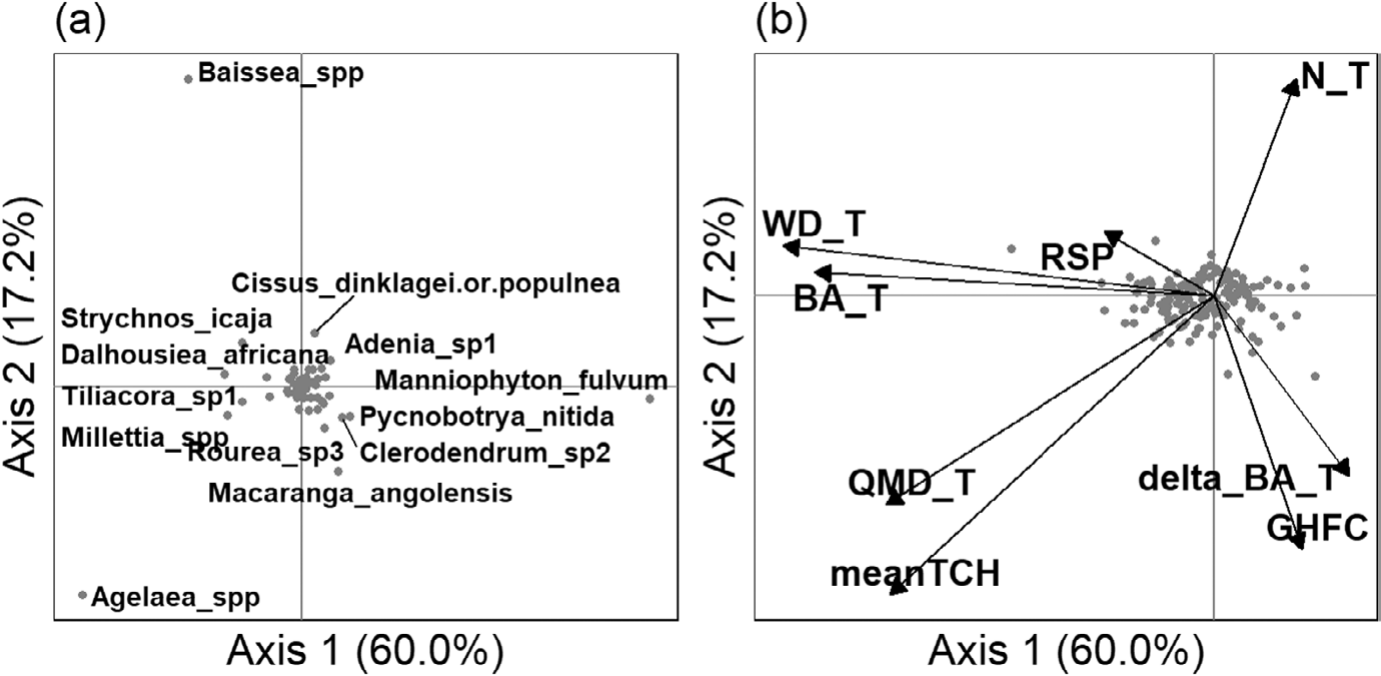
The relationship between liana floristic composition and the environment assessed through a non‐symmetric canonical correspondence analysis (NSCAIV). (a) Projection of liana species scores (in gray dots) on the first two axes of the NSCAIV. (b) Projection of environmental variables on the first two axes of the NSCAIV (in gray dots). The environmental variables are represented by delta_BA_T = tree basal area change, meanTCH = mean canopy height, GHFC = giant herbs foliar cover, QMD_T = tree quadratic mean diameter, BA_T = tree total basal area, N_T = tree abundance, WD_T = tree mean wood density, and RSP = relative slope position.

Overall, environmental variables explained 9.9% and 7.0% of the liana floristic variation with the NSCAIV (*p*‐value = 0.045) and PCCA (*p*‐value = 0.003) analyses and led to qualitatively similar results (Figure 4 and Appendix S1: Supplementary 7), as follows. The first axis opposed forests with tall canopies composed of large and hardwood trees to forests with shorter canopies and light‐wooded trees. The second axis opposed forests with a relatively high abundance of small trees to forests dominated by giant herbs, where a larger deficit of small trees occurred, but a higher increase in tree basal area was observed. Because the PCCA overemphasized very rare species (≤ 2 individuals; Appendix S1: Supplementary 7), as expected for such analyses (Pélissier et al. 2003), we hereafter only refer to the NSCAIV results for the taxonomic composition (Figure 4). Along the first axis of the NSCAIV, species such as *Manniophyton fulvum* and *Pycnobotrya nitida* were associated with open canopies, while *Agelaea spp*. tended to occur in tall and dense canopy forests. The second axis of the NSCAIV suggested that *Macaranga angolensis* preferentially co‐occurred with giant herbs, while *Baissea spp*. tended to avoid them and to occur in areas with a greater number of small tree stems (Figure 4).

### 3.3 Liana Functional Composition

Overall, we found significant relationships between three liana functional traits and three environmental variables (Table 3). Only local forest structure variables influenced the functional composition of lianas. Liana species with high TD but low %PO_4_ values tended to increase their relative abundance in tall, dense canopy forests with large basal areas. We also found a weak but significant increase in the abundance of liana species with high SLA values in areas dominated by small‐sized trees. Local topography (RSP) did not affect liana community functional composition.

**TABLE 3:** Fourth‐corner correlations (N2w) between functional traits and environmental variables.

	%C	%N	%PO_4_	%K	SLA	Thick	TD
BA_T_	0.06	0.00	−0.07*	−0.03	−0.04	0.00	0.10*
QMD_T_	0.02	−0.02	−0.05	−0.01	−0.06*	0.03	0.07
N_T_	0.03	0.00	0.00	0.00	0.04	−0.04	0.01
ΔBA_T_	0.01	−0.03	0.00	0.00	−0.01	0.04	−0.01
WD_T_	0.03	0.00	−0.08	−0.01	−0.06	0.00	0.06
meanTCH	0.07	−0.03	−0.10*	−0.06	−0.05	0.02	0.14*
GHFC	−0.05	0.00	0.05	0.00	0.01	0.01	−0.04
RSP	0.00	−0.02	0.00	0.01	−0.02	0.02	0.00

*Note:* Significant correlations, obtained with 999 permutations, are reported in red and blue for negative and positive correlations, respectively. Values in bold and with an * represents max *p*‐values < 0.05.

Abbreviations: ΔBA_T_, tree basal area change; BA_T_, tree total basal area; GHFC, giant herbs foliar cover; meanTCH, mean canopy height; N_T_, tree abundance; QMD_T_, tree quadratic mean diameter; RSP, relative slope position; SLA, liana specific leaf area; TD, liana tissue density; Thick, liana leaf thickness; WD_T_, tree mean wood density.

We found no significant variation of liana CWM traits along the two main liana floristic gradients (NSCA scores), except for liana TD (*p*‐value = 0.040; Appendix S1: Supplementary 8). Interestingly, the residuals of the NSCAIV for lianas, i.e., the variation in liana floristic composition that could not be explained by the studied environmental variables, were still marginally significantly correlated with the mean liana TD (*p*‐value = 0.057; Appendix S1: Supplementary 8).

### 3.4 Liana‐Host Specificity

The floristic composition of lianas varied significantly with the floristic composition of the tree community (RV test observation = 0.11, *p*‐value = 0.002), even after removing the effects of the environmental variables (RV test observation = 0.097, *p*‐value = 0.037). The randomization procedure revealed that the 10 most abundant liana taxa had significant host specificity: 19% of pairwise associations with the 24 most abundant tree species were significant (*p* < 0.05), with 23 attraction and 22 avoidance relationships (Table 4). In most cases, liana species exhibited both attraction and repulsion patterns with tree species (e.g., *M. fulvum* ), with obvious similarities among lianas: some tree species tend to either attract systematically (e.g., *Cleistanthus caudatus, Gilbertiodendron dewevrei, Leplaea thompsonii, Pentaclethra macrophylla, Petersianthus macrocarpus*) or escape liana infestation (e.g., *Celtis mildbraedii, Dichostemma glaucescens, Greenwayodendron suaveolens, Staudtia kamerunensis var. gabonensis*). Two tree species (*Diospyros bipindensis and Manilkara mabokeensis*) tended to both attract and avoid some liana species, but without regard to their climbing mechanisms. Overall, there were no obvious relationships between liana climbing mechanisms and tree species preferences. Only one tree species, *Gilbertiodendron dewevrei*, tended to attract lianas with the same climbing mechanism (active mechanisms).

**TABLE 4:** Results from the host‐species randomization test for the 10 most abundant liana species (*N* ≥ 50 individuals) and the 24 most abundant tree species (*N* ≥ 100 individuals).

		Liana species									
		Agelaea spp (A)	Baissea spp (A)	Dalhousiea africana (A)	Loeseneriella spp (A)	Manniophyton fulvum (P)	Pycnobotrya nitida (P)	Strychnos icaja (P)	Strychnos spp (P)	Tiliacora sp1 (A)	Triclisia macrophylla (A)
Tree species	Angylocalyx pynaertii	2 [0; 6]	2 [1; 9]	2 [2; 10]	2 [0; 3]	0 [3; 12]	0 [0; 2]	2 [0; 4]	0 [0; 4]	0 [0; 3]	1 [0; 3]
	Carapa procera	2 [1; 9]	2 [1; 9]	7 [5; 16]	1 [0; 4]	17 [9; 24]	0 [0; 4]	0 [0; 5]	2 [0; 6]	0 [0; 3]	1 [0; 4]
	Celtis mildbraedii	0 [0; 7]	4 [2; 11]	1 [3; 12]	0 [0; 3]	2 [5; 16]	0 [0; 2]	0 [0; 4]	0 [0; 5]	0 [0; 4]	1 [0; 4]
	Cleistanthus caudatus	23 [5; 15]	18 [7; 19]	36 [14; 29]	6 [0; 7]	36 [21; 37]	5 [1; 8]	11 [1; 8]	4 [1; 9]	1 [0; 6]	1 [0; 5]
	Dichostemma glaucescens	7 [5; 17]	12 [13; 28]	13 [16; 34]	2 [1; 9]	29 [39; 62]	4 [1; 7]	0 [1; 9]	6 [2; 12]	2 [0; 6]	4 [1; 8]
	Diospyros bipindensis	17 [4; 15]	17 [6; 19]	12 [13; 30]	1 [0; 6]	23 [19; 37]	0 [0; 4]	1 [0; 5]	4 [1; 9]	0 [0; 7]	1 [0; 6]
	Diospyros iturensis	3 [1; 8]	10 [2; 11]	10 [5; 16]	0 [0; 4]	4 [7; 20]	1 [0; 3]	0 [0; 5]	3 [0; 5]	0 [0; 5]	0 [0; 3]
	Garcinia punctata	6 [1; 10]	5 [3; 14]	13 [6; 18]	0 [0; 5]	17 [10; 24]	2 [0; 5]	3 [0; 6]	2 [0; 5]	0 [0; 4]	0 [0; 4]
	Gilbertiodendron dewevrei	9 [1; 9]	5 [1; 8]	28 [7; 19]	5 [0; 4]	19 [8; 21]	2 [0; 5]	5 [0; 6]	7 [1; 7]	11 [1; 8]	7 [0; 4]
	Grossera macrantha	1 [0; 7]	3 [2; 10]	8 [3; 13]	1 [0; 3]	20 [5; 16]	2 [0; 3]	0 [0; 5]	2 [0; 4]	0 [0; 4]	0 [0; 4]
	Leplaea thompsonii	13 [1; 8]	23 [3; 12]	33 [5; 16]	6 [0; 5]	39 [10; 24]	0 [0; 4]	0 [0; 4]	9 [0; 6]	1 [0; 3]	2 [0; 4]
	Macaranga spinosa	2 [0; 7]	6 [2; 10]	7 [4; 15]	1 [0; 4]	15 [13; 28]	4 [0; 4]	0 [0; 4]	4 [0; 6]	0 [0; 3]	2 [0; 5]
	Manilkara mabokeensis	5 [1; 9]	0 [3; 13]	5 [6; 18]	1 [0; 5]	4 [10; 23]	4 [0; 3]	3 [0; 7]	1 [0; 6]	2 [0; 4]	2 [0; 5]
	Nesogordonia kabingaensis	1 [0; 6]	3 [1; 9]	2 [2; 10]	0 [0; 3]	2 [5; 16]	0 [0; 2]	1 [0; 4]	1 [0; 5]	0 [0; 3]	0 [0; 3]
	Pancovia harmsiana	2 [1; 8]	3 [1; 9]	8 [3; 13]	0 [0; 3]	5 [6; 17]	0 [0; 3]	1 [0; 4]	1 [0; 4]	1 [0; 2]	0 [0; 3]
	Pancovia laurentii	1 [0; 6]	10 [1; 9]	5 [2; 12]	0 [0; 3]	12 [5; 15]	1 [0; 2]	0 [0; 3]	1 [0; 4]	1 [0; 2]	0 [0; 3]
	*Pentaclethra macrophylla*	7 [1; 8]	4 [1; 9]	24 [4; 14]	3 [0; 4]	43 [7; 18]	1 [0; 3]	7 [0; 4]	2 [0; 5]	2 [0; 4]	4 [0; 4]
	Petersianthus macrocarpus	1 [1; 7]	12 [1; 10]	5 [3; 14]	0 [0; 4]	16 [6; 17]	1 [0; 4]	8 [0; 3]	1 [0; 5]	0 [0; 3]	2 [0; 4]

	Greenwayodendron suaveolens	2 [2; 12]	8 [5; 16]	6 [8; 21]	5 [0; 6]	3 [10; 24]	2 [0; 3]	1 [0; 6]	2 [0; 6]	1 [0; 5]	1 [0; 5]
	Quassia silvestris	1 [0; 6]	2 [1; 8]	3 [2; 12]	0 [0; 3]	1 [6; 17]	0 [0; 3]	0 [0; 4]	0 [0; 4]	0 [0; 3]	0 [0; 3]
	Rinorea oblongifolia	3 [0; 6]	1 [1; 9]	4 [3; 12]	0 [0; 3]	1 [5; 16]	1 [0; 4]	2 [0; 3]	1 [0; 4]	0 [0; 3]	1 [0; 3]
	Santiria trimera	7 [0; 6]	5 [0; 7]	9 [2; 10]	1 [0; 3]	13 [4; 13]	0 [0; 3]	1 [0; 3]	1 [0; 4]	3 [0; 3]	0 [0; 3]
	Staudtia kamerunensis var. gabonensis	1 [2; 10]	4 [3; 14]	5 [7; 20]	0 [0; 4]	11 [13; 27]	2 [0; 3]	1 [0; 6]	2 [0; 6]	1 [0; 5]	0 [0; 5]
Strombosia pustulata	1 [1; 8]	5 [2; 11]	4 [2; 11]	1 [0; 4]	1 [4; 13]	0 [0; 2]	0 [0; 3]	1 [0; 5]	1 [0; 3]	0 [0; 3]

*Note:* Liana taxa with (A) an active climbing mechanism and taxa with (P) a passive one. The observed number of lianas infesting the focal tree species is presented, with its confidence interval obtained from 999 simulations of liana–tree associations (in square parenthesis). Significant attraction and repulsion relationships are illustrated in blue and red, respectively.

## 4 Discussion

In this study, we used a combination of ground inventories, functional traits, and high‐resolution drone imagery to understand how local forest structure, tree composition, and topography influence the structure, floristic, and functional composition of liana communities. Consistent with our hypothesis (H1), and with previous studies mostly conducted in the Neotropics, forest structure was the primary factor affecting liana abundance, with higher abundances and total basal area in open canopies. We also provide evidence that lianas and giant herbs mutually exclude each other in open‐canopy areas. The floristic composition of lianas was not spatially structured at the studied scale and was only weakly influenced by environmental variables compared to our expectations (H2). Local topography, though relatively unchanged at the large scale but featuring marked small‐scale features such as termite mounds, had no discernible effect on liana structure nor functional composition. In contrast, tall and dense canopy forests appeared to be associated with a higher proportion of large lianas with more conservative traits (higher TD and lower %PO_4_ and SLA values), supporting our hypothesis on functional composition (H2). After ruling out the environmental influences on liana and tree communities and contrary to our expectations (H3), we found significant covariation between their composition and a randomization approach confirmed a higher degree of host specificity than expected by chance. We discuss these results and their implications below.

### 4.1 Gap Hypothesis and Structure Shifts Along Local Forest Structure

We showed that the structure of liana communities is tightly associated with the structure of tree communities and the presence of giant herbs. In particular, liana abundance (stem density) was found to be the most predictable metric (38% of the variance explained). We specifically found higher abundances in areas characterized by smaller and light‐wooded trees and a low canopy height, corresponding to open‐canopy areas. These results are consistent with those of previous studies, mostly conducted in the Neotropics, which validated the so‐called “gap hypothesis,” i.e., that disturbance and gaps in old‐growth forest canopies promote liana abundance (Ewango et al. 2015; Ledo and Schnitzer 2014; Schnitzer 2018; Schnitzer et al. 2012, 2021; Schnitzer and Bongers 2011; Villagra et al. 2021). Indeed, lianas have been found to proliferate in response to increased light availability in forest gaps following a disturbance, primarily through high clonal reproduction (Ledo and Schnitzer 2014). This proliferation significantly reduces tree regeneration and diversity (Schnitzer et al. 2000; Schnitzer and Carson 2010). Moreover, lianas can persist in forest gaps over time, especially in regions characterized by a high temperature and climatic water deficit (Ngute et al. 2024). A recent study from Southeast Asia even showed that liana abundance increased continuously along a successional gradient (Lomwong et al. 2023). Combined with the gap hypothesis, this result suggests that lianas can recruit continuously along secondary succession and then maintain their abundance through the gap dynamics of old‐growth forests. Such an increase in abundance along secondary succession was interpreted as the consequence of different niches and light requirements of liana species, with an increasing abundance of shade‐tolerant species in older growth forests with lower light conditions (Lomwong et al. 2023). Indeed, ecological strategies are known to differ among liana species (Putz and Mooney 1991), leading to different light requirements and thus responses to changing light conditions (Yuan et al. 2016), an explanation also supported by our results through different functional compositions of lianas in canopy openings and close dense canopy areas (see discussion point 4.4). In addition, we found that lianas tended to be significantly larger in areas characterized by a larger total tree basal area and a smaller number of trees belonging to hardwood species. Poulsen et al. (2017) found consistently in a forest in Gabon that the abundance of large lianas increased with the abundance of large trees and with the mean wood density of tree communities, both of which are characteristics of old‐growth forests. Letcher and Chazdon (2009) also showed that the highest values of liana biomass (estimated using diameter‐based allometry) were at sites with old‐growth trees in successional chronosequences in Costa Rica. The proportion of large‐diameter liana stems may indeed indicate different stages of forest succession, as suggested by Dewalt et al. (2000), who demonstrated a decrease in liana abundance with stand age but an increase in total basal area. In our study, we also find an increase in the quadratic mean diameter of lianas with tree basal area and wood density but a decrease in the total basal area of lianas, i.e., the large number of small liana stems in open‐canopy areas compensates for their small individual basal area. Finally, Lomwong et al. (2023) found an additional pattern of variation in stem size and abundance in a forest in Thailand, with an increase in both liana abundance and basal area with aging forest succession. Overall, these results suggest that there are different patterns of change in liana community structure along environmental gradients and call for more research about the mechanisms behind these patterns, particularly exploring the functional changes related to different light requirements of lianas.

### 4.2 Competition With Giant Herbs

Our results suggest strong competition between woody lianas and giant herbs (Commelinales and Zingiberales) in open‐canopy areas. Among the dominant giant herbs in the study area was the climbing species *Haumania danckelmaniana* J. Braun & K. Schum. Milne‐Redh. Giant herbs are known to be very light‐demanding and to rapidly preempt above‐ and below‐ground resources, drastically limiting woody plant regeneration (Pouteau et al. 2024). Our study suggests that giant herbs in central Africa, and perhaps elsewhere, constitute a fourth potential pathway for gap‐phase regeneration, in addition to other life forms such as trees, palms, and lianas (Letcher 2015; Schnitzer et al. 2000).

Facilitation between different climbing species is known to occur when lianas and other climbers use each other as support to reach the canopy (Putz 1984a, 1984b). The reason why facilitation between woody and nonwoody climbers did not occur in our study area remains unclear. Two possible explanations for this remain to be examined. First, facilitation between lianas has been observed mainly in large lianas, which support smaller lianas (Campanello et al. 2007). At our study site, *H. danckelmaniana* barely reached 2 cm in diameter and rarely reached long distances (up to 15 m). However, while the size of the supporting species might limit the ultimate height that can be reached, most lianas climb small‐sized supports to begin their upward growth (Lehnebach et al. 2022). Second, giant herbs are known to rapidly develop multi‐shade leaf layers that drastically reduce light availability and thus seed germination (Brncic 2002). Further detailed work should focus on understanding the conditions under which successional processes might branch toward a liana‐ or giant herb‐dominated pathway.

### 4.3 Hyperdominance in the Liana Flora

Consistent with previous studies from central Africa (Ewango et al. 2015; Mumbanza et al. 2020), we found marked hyperdominance in the liana flora, with more than half of the individuals belonging to only three taxa. Our site had more or less the same abundant genera and families as distant African sites (Ewango et al. 2015; Mumbanza et al. 2020; Parren 2003; Thomas et al. 2014), suggesting that these abundant taxa exhibit a regional hyperdominance. *Manniophyton fulvum* (Euphorbiaceae) was by far the most abundant species, accounting for nearly one‐third of the lianas at our study site. It is a geographically widespread species (Borsch et al. 2020) and found to be the most dominant species in two other studies in the Democratic Republic of Congo (DRC; Ewango et al. 2015; Mumbanza et al. 2020). Although these two studies considered this species to be a habitat generalist, we found that it was associated with open‐canopy and short‐stature forests. Another example is the pantropical genus *Strychnos*, which was reported to be the most abundant genus in Korup National Park in Cameroon (Thomas et al. 2014) and was the third most abundant genus at our study site, where it appeared to be a habitat generalist. Taken together, these results suggest that both the local and regional central African liana flora are dominated by a few dominant and widespread taxa, even more than for tropical trees (Bastin et al. 2015; Ter Steege et al. 2013).

### 4.4 Weak but Significant Floristic and Functional Shifts Along Local Forest Structure Gradients

Our results suggested that the floristic composition of lianas does not vary at a larger scale than the quadrat in our study site. We specifically found that the local environmental variables had only a weak but significant effect (9.9%) on liana floristic composition. Consistently, in the DRC, weak floristic composition changes between regrowth and old‐growth mixed forests were reported (Mumbanza et al. 2020). Moreover, at a site in central Thailand, liana species assemblages were also found to be only weakly structured by local environmental variables (Chanthorn et al. 2024), but liana species composition differed strongly between forest succession stages (Lomwong et al. 2023).

We only found a few significant shifts in liana functional traits along environmental gradients in our study area. Liana stem TD increased, and leaf phosphorus concentration (%PO_4_) and SLA weakly decreased in taller and denser canopies. These results are consistent with those of other studies on liana traits along a forest successional gradient (Mumbanza et al. 2022; Villagra et al. 2021). SLA and %PO_4_ are commonly measured leaf traits for which greater values indicate faster resource acquisition and growth (Baraloto et al. 2010; Reich 2014; Rüger et al. 2012; Wright et al. 2004), whereas higher wood density indicates higher investment in stem structure, shown to confer greater resistance to water stress and disturbance in trees (Chave et al. 2009; Van Gelder et al. 2006). Therefore, as observed in trees, liana community composition shifts along the successional gradient from communities dominated by rapid resource acquisition strategies to conservative strategies, demonstrating that the liana flora exhibits, to an extent, a range of strategies adapted to different habitats (Letcher 2015; Liu et al. 2021a, 2021b; Rocha et al. 2022). Most other traits were not significantly related to the environmental variables studied, and we did not detect any trait shifts along the local topographical gradient (RSP) indicating that termite mounds do not impact liana assemblages. Surprisingly, the main floristic gradients in the study area were not significantly associated with functional composition, except for TD, even when the effect of the environment was removed.

The importance of liana TD along floristic gradients suggests that stem traits play an important role in their ecological strategies. The tissue organization, mechanics, and wood density of lianas are much more complex than those of trees, with a wide diversity of vascular configurations (Darwin, 1875; Caballé, 1993; Isnard & Silk, 2009; Angyalossy et al. 2015). This diversity may translate into different functions and/or solutions for dealing with different environmental conditions (Rocha et al. 2022). Thus, considering macro‐anatomical wood characteristics seems to be a promising way to better understand the life history of liana species and decipher functional relationships with the environment.

### 4.5 Host‐Species Specificity

The existence of host‐species‐specific relationships between trees and lianas has been a long‐standing question in liana ecology and, although relatively understudied, has led to conflicting results (Heymann et al. 2022; Song et al. 2023; Uwalaka et al. 2021; Vivek and Parthasarathy 2016). Our co‐inertia analysis revealed significant covariation between liana and tree community compositions, even after accounting for underlying environmental gradients. This result either suggests that (i) there is liana‐host specificity or (ii) our analysis failed to account for environmental variables that influenced both liana and tree compositions. Our randomization procedure confirmed the first hypothesis, with 19% significant host‐specific relationships between the most abundant liana and tree species. Although some studies have suggested that there were no species‐specific relationships between trees and lianas (Vivek and Parthasarathy 2016), others have found significant relationships (Heymann et al. 2022; Song et al. 2023; Uwalaka et al. 2021). In addition, some studies examining liana infestation from the point of view of trees have found that tree species identity is an important factor explaining liana species distributions, significantly more so than environmental variables (Nesheim and Økland 2007). More specifically, Nesheim and Økland (2007) found that environmental variables, such as canopy openness and light availability, played an important role in the early life stages of lianas prior to their attachment to host trees, while host tree characteristics played a greater role after initial attachment. The morphological and architectural characteristics of host trees, such as bark type, tree size, or length of branch‐free trunks, were shown to affect liana colonization, probably through specific liana life‐history traits, such as reaching support, attachment, and climbing mechanisms (Addo‐Fordjour and Afram 2021; Campanello et al. 2007; Carsten et al. 2002; Heijden et al. 2008; Nesheim and Økland 2007; Uwalaka et al. 2021). For example, in our study, *Staudtia kamerunensis var. gabonensis*, which is characterized by exfoliating bark, tends to repel all the studied liana species. However, Song et al. (2023) did not provide sufficient support for the role of tree bark type in liana infestation prevalence. Regarding liana morphology, a recent study showed that tendril climbers tended to have short reach distances to find host trees, species with hooks and prehensible branches had longer reaches, and twining species displayed the largest range of reach distances (Hattermann et al. 2022). The abundance of twining liana species has also been found to decrease with forest succession (Dewalt et al. 2000; Letcher and Chazdon 2012), and shifts in tree composition have been found to influence liana species distribution in relation to their climbing mechanisms (Bongers et al. 2020). However, we found no apparent relationship between the climbing mechanisms of the most abundant liana species and tree species identity. Further studies are needed to better determine and understand how liana and tree characteristics interact to create host‐specific relationships.

## 5 Conclusion

Our results showed that liana community structure was mainly determined by local forest structure but that its floristic and functional composition varied weakly along local environmental gradients; however, faster acquisition strategies tended to occur in open‐canopy areas. This study thus provided further evidence for the neotropical gap hypothesis in a central African forest, but showed that the increase of lianas in canopy openings is only weakly associated with functional changes. We also provided the first evidence for a marked competition between lianas and giant herbs and revealed that the species composition of trees and lianas exhibited significant species‐specific associations for the most common lianas and trees.

Overall, our results open up two promising avenues for a better understanding of the spatiotemporal distribution of liana communities. First, research on the functional ecology of lianas should be pursued to identify traits that best reflect their life history. Here, we highlighted the importance of liana stem TD for liana distribution, suggesting that generating more detailed wood traits would be promising. Second, we identified significant host‐specific associations between liana and tree species, but failed to understand the causes of this host specificity. Larger‐scale observations or experiments could confirm these important results and provide important insights into the underlying mechanisms. We believe that these two research avenues may significantly contribute to a better understanding of liana ecology and thus to a better prediction of the impact of lianas at the ecosystem level.

## Author Contributions

**Begüm Kaçamak:** conceptualization (lead), data curation (lead), formal analysis (lead), methodology (lead), writing – original draft (lead), writing – review and editing (lead). **Maxime Réjou‐Méchain:** conceptualization (equal), data curation (equal), formal analysis (equal), methodology (equal), resources (equal), writing – original draft (equal), writing – review and editing (equal). **Nick Rowe:** resources (equal), writing – original draft (equal), writing – review and editing (equal). **Vivien Rossi:** writing – review and editing (equal). **Nicolas Barbier:** data curation (equal), writing – review and editing (equal). **Samantha Bazan:** data curation (equal), writing – review and editing (equal). **Eric Forni:** data curation (equal), writing – review and editing (equal). **Daniel Guibal:** data curation (equal), writing – review and editing (equal). **David J. Harris:** data curation (equal), writing – review and editing (equal). **Grace Jopaul Loubota Panzou:** writing – review and editing (equal). **Jean‐Joël Loumeto:** writing – review and editing (equal). **Eric Marcon:** writing – review and editing (equal). **Bruno X. Pinho:** writing – review and editing (equal). **Isaac Zombo:** data curation (equal), writing – review and editing (equal). **Sylvie Gourlet‐Fleury:** conceptualization (equal), data curation (equal), resources (equal), writing – review and editing (equal).

## Conflicts of Interest

The authors declare no conflicts of interest.

## Supporting information


Appendix S1.

## Data Availability

Data are available in the IRD Dataverse (https://doi.org/10.23708/5H5IXX). This dataset contains the liana plot‐level information about the different data used in the paper. Liana structure metrics are at the liana quadrats (20 × 20 m^2^) level. Environmental metrics are at the liana quadrat level with a 10‐m buffer on each side (40 × 40 m^2^). Liana functional trait metrics are community‐weighted means at the quadrat level.
