# Supplementary material for: Local Forest Structure and Host Specificity Influence Liana Community Composition in a Moist Central African Forest
Source: Ecol Evol. 2025 Mar 11;15(3):e71075. doi: 10.1002/ece3.71075 (PMC11896882; doi:10.1002/ece3.71075)

# Supplementary

[Supplementary 1. Genetic analyses 1](#_Toc177464839)

[Supplementary 2. Termite mounds within the permanent plots in the study site. 2](#_Toc177464840)

[Supplementary 3. Relative slope position index derived from lidar-based elevation model over the study area and capturing well the termite mounds (high values in red). 3](#_Toc177464841)

[Supplementary 4. Plan 1-2 of a Non-Symmetrical Correspondence Analysis (NSCA) performed on 82 liana species with species distribution (in grey dots). 3](#_Toc177464842)

[Supplementary 5. Spatial structure in floristic composition on the 144 liana quadrats and their spatial variogram. (a) Spatial distribution of the color-coded scores of the second axis of the Non-Symmetric Correspondence Analysis (NSCA) of liana floristic composition in UTM zone 33N coordinates. There is no visible spatial pattern of the floristic composition of lianas (of the different colors). For illustrative purposes, a 10-m buffer has been added to the quadrat to better visualize the colors. (b) Insert graph representing the variogram of NSCA axis 2 scores, with 11 distance classes in the x axis and the variance in y axis. The dotted lines on the variogram represent the confidence intervals at 95% and the solid lines the observed variogram. The variogram stays inside the confidence interval for all distance classes and does not deviate from a random structure, confirming the absence of a spatial pattern of liana floristic composition. Individual P values for each distance class are reported in Fig. S6. 4](#_Toc177464843)

[Supplementary 6. Table of p-values for permutation tests (n=9999) at different distance classes for the variogram of the scores of NSCA axis 1 and axis 2. 5](#_Toc177464844)

[Supplementary 7. The relationship between liana floristic composition and the environment assessed through a partial canonical correspondence analysis (PCCA). Projection of environmental variables on the first two axes of the PCCA, with species with grey dots and liana quadrats with red crosses. The environmental variables are represented by delta_BA_T=tree basal area change, meanTCH=mean canopy height, GHFC=giant herbs foliar cover, QMD_T=tree quadratic mean diameter, BA_T=tree total basal area, N_T=tree abundance, WD_T=tree mean wood density, and RSP=relative slope position. 5](#_Toc177464845)

[Supplementary 8. Relationship between (a) the first axis of the NSCA and (b) the first axis of the residuals of the NSCAIV (i.e. the part of the floristic composition of lianas not explained by environmental variables), and liana tissue density. 6](#_Toc177464846)

Supplementary 1. Genetic analyses

DNA was successfully extracted from 192 dried liana leaf tissue samples, with at least one extraction per vernacular name, and then, focusing on samples for which taxonomic uncertainty was the highest or on genera known to contain several lianescent species. We sequenced two plastid genes, rbcl and matK, as these two loci have been identified as the best candidates for barcoding terrestrial land plants (CBOL Plant Working Group1, 2009. All DNA extractions were achieved using a Qiagen kit (QIAamp, 2010) and the Polymerase Chain Reaction (PCR) was performed following the protocol from Dunning and Savolainen (2010) for matK and rbcl. All isolates were sequenced in both directions (Forward and Reverse) using the Sanger method (Gonzalez et al., 2009). Consensus sequences from forward and reverse reads were built using the Geneious software (Geneious, 2018). Then, all sequences were aligned in the MEGA software (Kumar et al., 2018). Basic Local Alignment Search Tool (BLAST), an algorithm used to compare DNA sequences and identify similar sequences above a certain threshold was run through GenBank (Benson et al., 2008). We identified each specimen using first the BLAST results to restrict the identification to a given taxonomic group and then using taxa identification keys based on vegetative or fertile (when available) characters in the herbarium samples and other open-access herbarium databases.


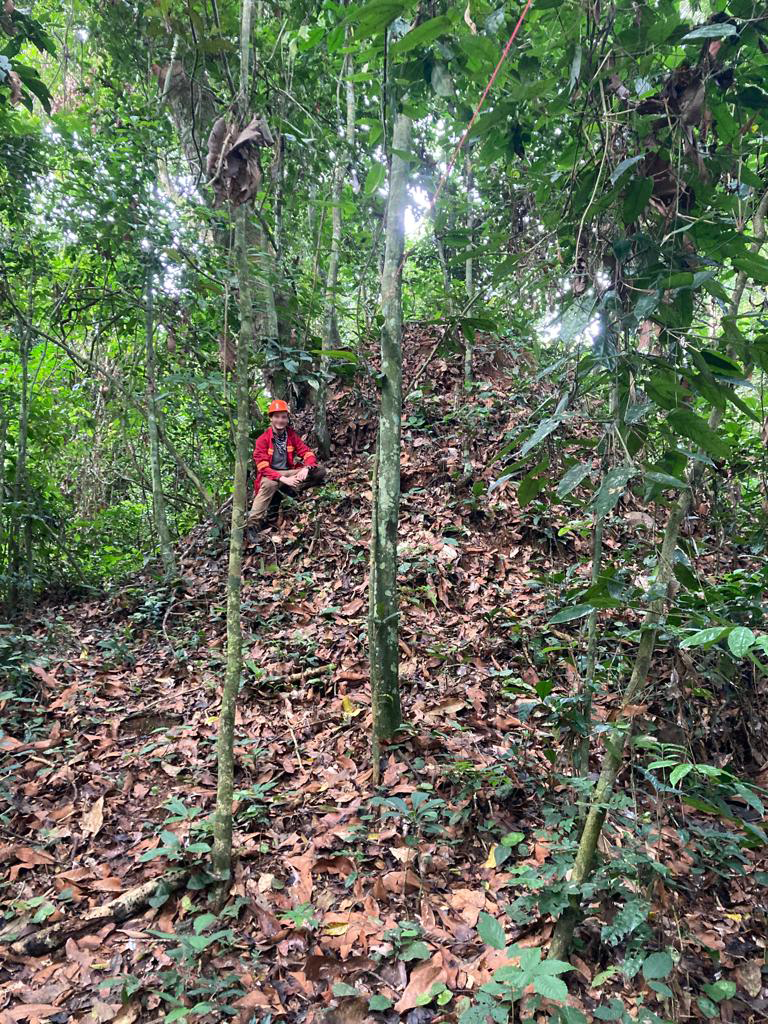
Supplementary 2. Termite mounds within the permanent plots in the study site.

Supplementary 3. Relative slope position index derived from lidar-based elevation model over the study area and capturing well the termite mounds (high values in red).


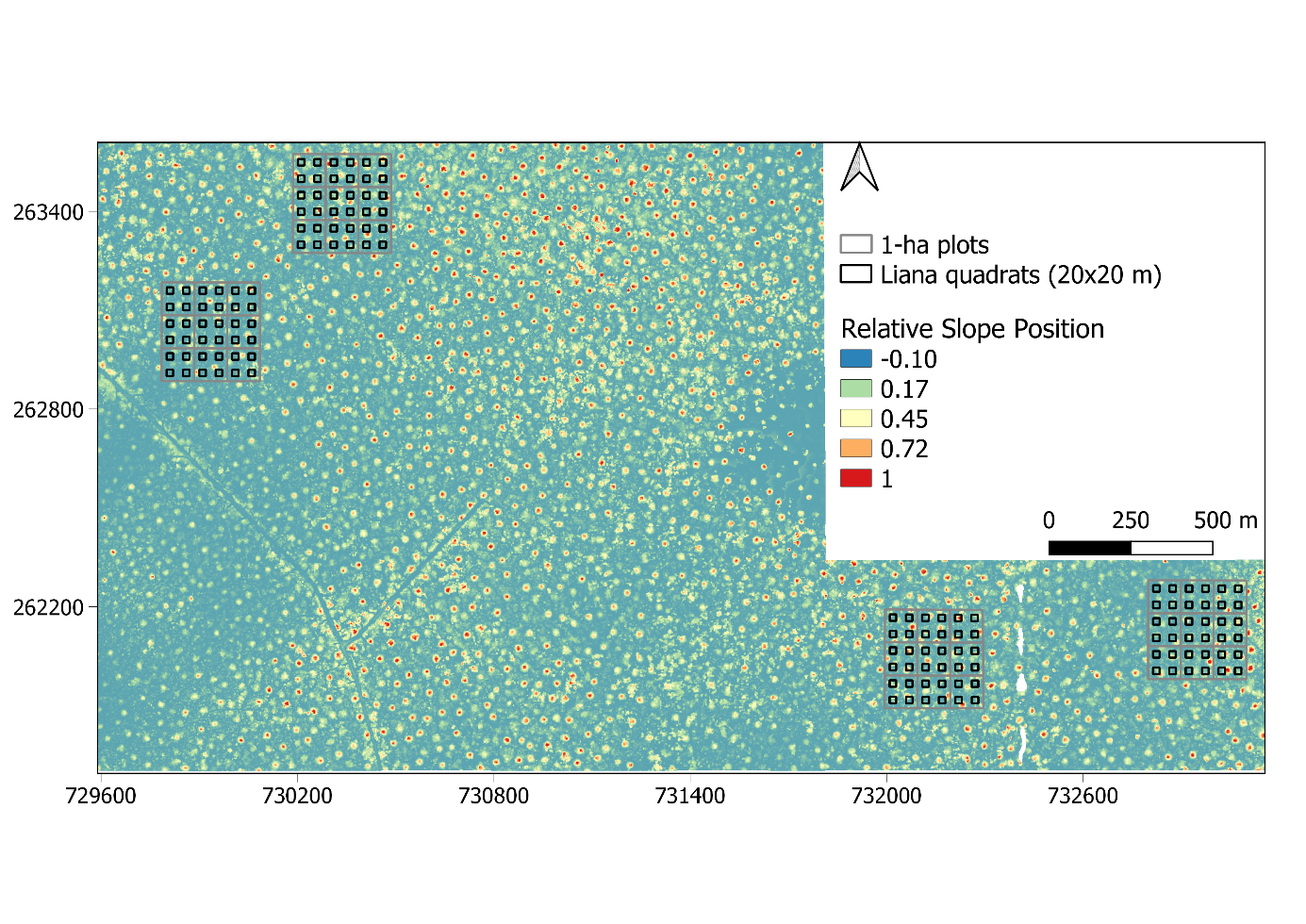


Supplementary 4. Plan 1-2 of a Non-Symmetrical Correspondence Analysis (NSCA) performed on 82 liana species with species distribution (in grey dots).


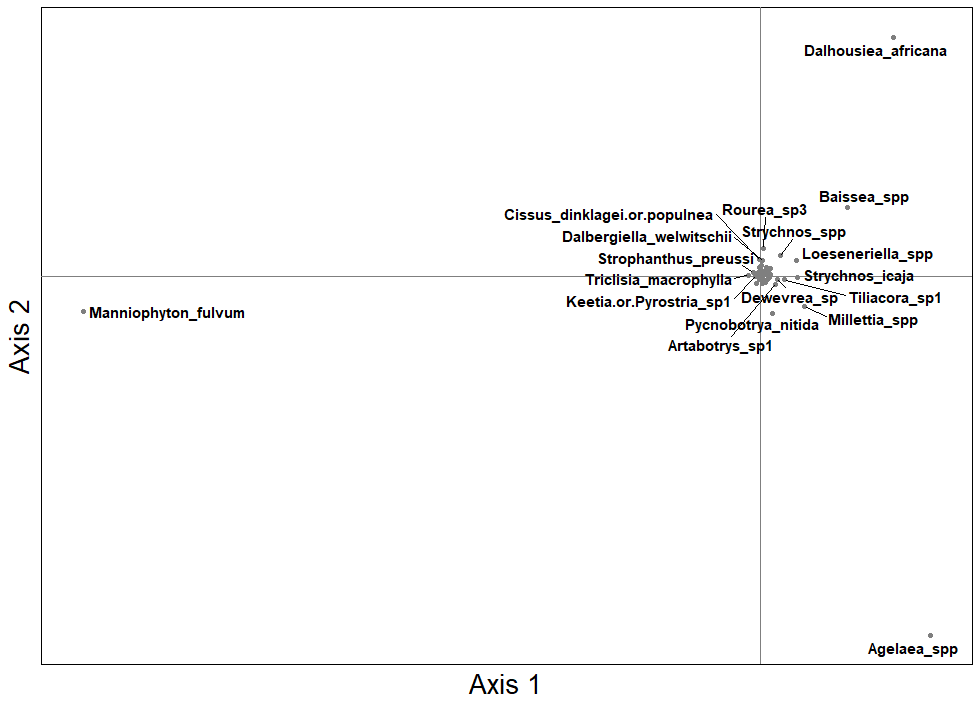


Supplementary 5. Spatial structure in floristic composition on the 144 liana quadrats and their spatial variogram. (a) Spatial distribution of the color-coded scores of the second axis of the Non-Symmetric Correspondence Analysis (NSCA) of liana floristic composition in UTM zone 33N coordinates. There is no visible spatial pattern of the floristic composition of lianas (of the different colors). For illustrative purposes, a 10-m buffer has been added to the quadrat to better visualize the colors. (b) Insert graph representing the variogram of NSCA axis 2 scores, with 11 distance classes in the x axis and the variance in y axis. The dotted lines on the variogram represent the confidence intervals at 95% and the solid lines the observed variogram. The variogram stays inside the confidence interval for all distance classes and does not deviate from a random structure, confirming the absence of a spatial pattern of liana floristic composition. Individual P values for each distance class are reported in Fig. S6.


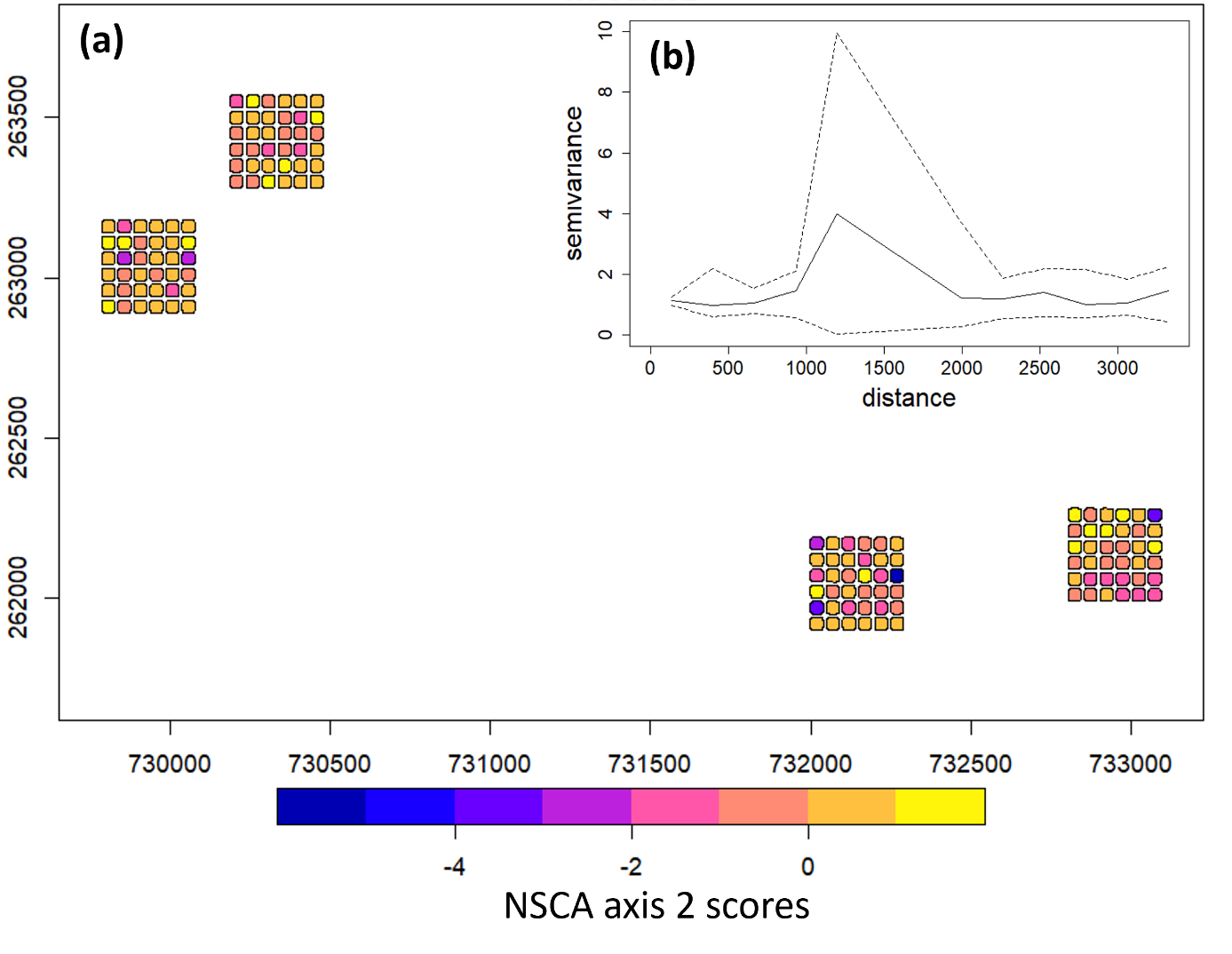


Supplementary 6. Table of p-values for permutation tests (n=9999) at different distance classes for the variogram of the scores of NSCA axis 1 and axis 2.

| Distance classes | 133 | 399 | 666 | 932 | 1198 | 1997 | 2263 | 2530 | 2796 | 3062 | 3328 |
| --- | --- | --- | --- | --- | --- | --- | --- | --- | --- | --- | --- |
| p-values  (NSCA axis 1) | 0.66 | 0.92 | 0.90 | 0.69 | 0.69 | 0.80 | 0.82 | 0.73 | 0.56 | 0.53 | 0.82 |
| p-values  (NSCA axis 2) | 0.50 | 0.50 | 0.50 | 0.50 | 0.73 | 0.50 | 0.50 | 0.50 | 0.50 | 0.50 | 0.50 |

Supplementary 7. The relationship between liana floristic composition and the environment assessed through a partial canonical correspondence analysis (PCCA). Projection of environmental variables on the first two axes of the PCCA, with species with grey dots and liana quadrats with red crosses. The environmental variables are represented by delta_BA_T=tree basal area change, meanTCH=mean canopy height, GHFC=giant herbs foliar cover, QMD_T=tree quadratic mean diameter, BA_T=tree total basal area, N_T=tree abundance, WD_T=tree mean wood density, and RSP=relative slope position.


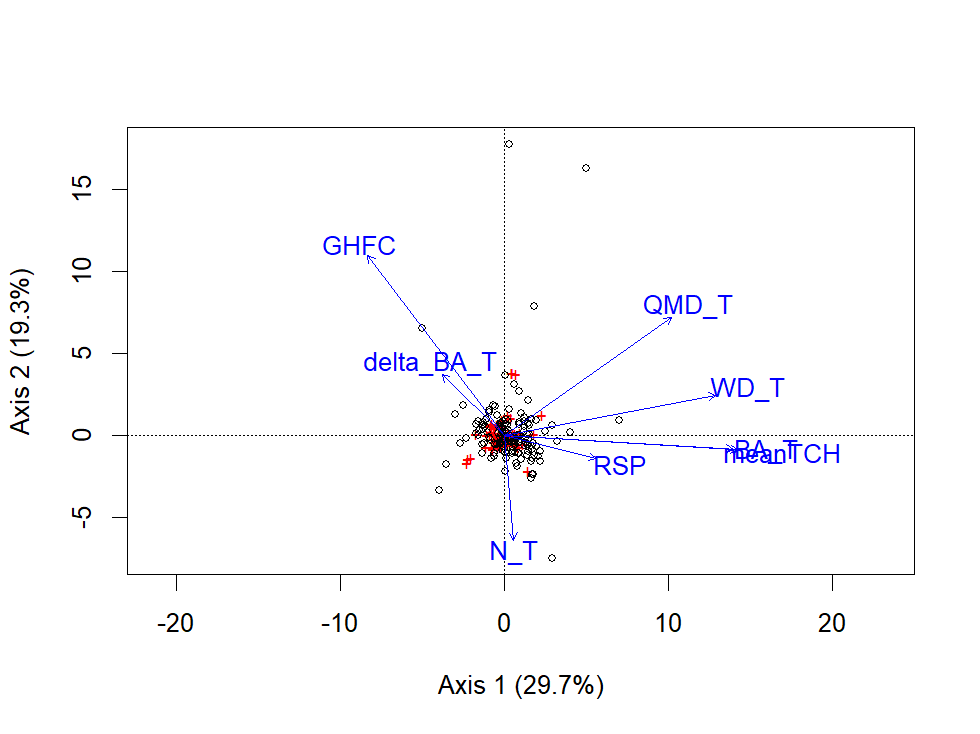


Supplementary 8. Relationship between (a) the first axis of the NSCA and (b) the first axis of the residuals of the NSCAIV (i.e. the part of the floristic composition of lianas not explained by environmental variables), and liana tissue density.


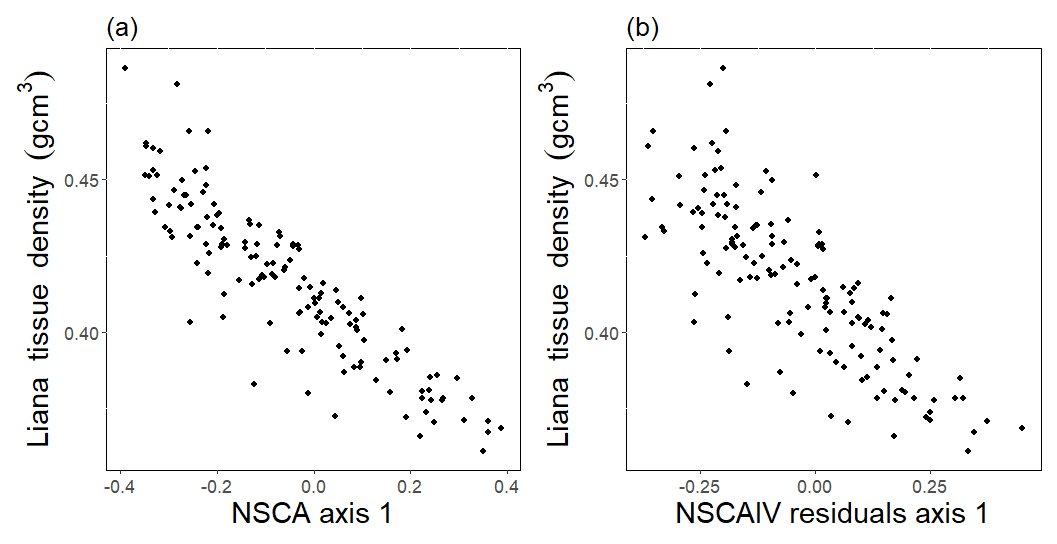

Supplement: Supplementary file 1 — Appendix S1. [file ECE3-15-e71075-s001.docx]
